# Simulation in Obstetric and Gynecologic Ultrasound Training: Design and Implementation Considerations

**DOI:** 10.3390/jcm14145064

**Published:** 2025-07-17

**Authors:** Sheldon Bailey, Christoph F. Dietrich, Jacqueline Matthew, Michael Bachmann Nielsen, Malene Roland Vils Pedersen

**Affiliations:** 1 School of Health and Life Sciences, Northern Alberta Institute of Technology, Edmonton, AB T5G 2R1, Canada; 2 Department of General Internal Medicine (DAIM), Hospitals Hirslanden Bern Beau Site, Salem and Permanence, 3013 Bern, Switzerland; c.f.dietrich@googlemail.com; 3 Department of Early Years Imaging, Kings College London, St Thomas Hospital, London SE1 7EH, UK; 4 Department of Radiology, Copenhagen University Hospital, Rigshospitalet, 2100 Copenhagen, Denmark; 5 Department of Clinical Medicine, University of Copenhagen, 2100 Copenhagen, Denmark; 6 Department of Radiology, University Hospital of Southern Denmark, 7100 Vejle, Denmark; 7 Department of Regional Health Research, University of Southern Denmark, 5230 Odense, Denmark; 8 Discipline of Medical Imaging & Radiation Therapy, University College Cork, T12 K8AF Cork, Ireland

**Keywords:** simulation, diagnostic, radiology, gynecology, phantoms, ultrasound

## Abstract

**Background/Objectives**: The use of simulation has become more popular in healthcare settings, and simulation is also very popular in ultrasound training, allowing the learners to virtually practice and improve clinical skills. Obstetric pathology and gynecologic lesions can have a large range of sonographic features, and the detection rates for these can be increased by using ultrasound simulation systems to train users. In the following paper, we provide insight into the application of simulation tools in obstetric and gynecologic ultrasound training. **Methods**: We present different ultrasound models for GYN/OB ultrasound training. **Results**: Ultrasound simulation is a key component of obstetrics and gynecology (OB/Gyn) ultrasound education. **Conclusions**: By examining the best practices, we highlight the diverse simulation options available to help learners technical and non-technical skills in a controlled learning environment.

## 1. Introduction

Ultrasound has become a very necessary tool in the detection and management of gynecologic disease and is even considered the gold-standard for routine obstetric imaging [1]. Being relatively inexpensive, portable, and not producing ionizing radiation, ultrasound imaging can be safely and readily implemented in many areas where diagnostic imaging is required. Additionally, technological advances in recent years, including the advent of contrast imaging and more accurate Doppler imaging techniques, has resulted in the application of this modality becoming more common in high-risk and interventional obstetrics and gynecology (OB/Gyn) cases [2]. The increased prevalence of this versatile modality has resulted in ultrasound being used in a high number of medical settings. While sonographers and radiologists maintain their presence as experts in the use and interpretation of medical ultrasound, a rising number of general medical practitioners, nurses, and midwives are also performing ultrasound examinations as a means of improving point-of-care patient assessments and managing wait times for routine examinations [3,4]. This diversity in practitioner background therefore requires deliberate efforts to standardize training and promote consistent accuracy in practice. This paper synthesizes current evidence to guide educators in building sustainable OB/Gyn ultrasound simulation programs and provide a broad perspective for undertaking such initiatives.

Imaging, image acquisition, the interpretation of ultrasound images, and the use of this interpretation in making medical decisions represent the four levels of skills acquisition in ultrasound training [1]. The first and second levels require a hands-on approach to learning that is typically supported using patient volunteers and simulation activities. For these levels, learners are required to practice extensively if mastery is to be gained. If such a learning journey is adequately supported, then reliable and reproducible training methods must be implemented. This indicates a need for the intentional and thoughtful design of instructional opportunities. This paper presents important considerations to guide medical educators when designing simulation-based training programs for obstetric and gynecologic ultrasound scanning. Information sources were selected through a narrative search approach, combining relevant literature with the authors’ clinical experiences. Sources were included based on their relevance to the focus of the article, including relevance for obstetric and gynecologic ultrasound training programs, with a primary focus on practical considerations and educational value.

## 2. Background—Simulation-Based Training in Ultrasound

The word *simulation* indicates an amount of pretense or imitation surrounding a situation or a process [5]. In healthcare training programs, a simulation is meant to provide learners with the opportunity to develop technical skills in an environment that safely allows for mistakes and the repetition of actions [6]. Although the use of healthcare simulation can be traced back for centuries, the modern era of simulation only began in the second half of the 20th century [7]. Recent years have seen a markedly increased uptake in the use of simulation to support healthcare training programs [8,9], and while the reasons for this are varied, they are all justified by the flexibility and safety that simulation-based training (SBT) offers. When properly applied, SBT allows learners to practice and hone their skills in controlled settings with no risks to real patients.

Still, the many technological advances of recent years are also now allowing simulation to be effectively accessed in non-traditional learning environments based on the learners’ schedule. While simulation marvels, such as breathing and coughing manikins, can be found in most simulation labs, web-based simulations and gamified learning scenarios also provide flexible options for learners to encounter opportunities for learning. These tools are well-applied in many ultrasound training programs.

SBT in ultrasound can help the learner develop critical technical sonographic skills and facilitate a smoother transition from learner to practitioner by bridging the gap between the classroom and clinical practice. However, SBT also has the potential to help learners become more proficient in the more nuanced areas of their practice, necessarily complementing finite, technical skills with tools to better manage interactions with patients. For example, standardized patients (SPs), who are individuals trained to depict different types of patient scenarios, illnesses, and behavioral challenges [10], can be used to support this aspect of student training. This support the illustration of real-life scenarios that allow learners to develop clinical and interpersonal skills in a safe and controllable environment, while also providing opportunities for learners to begin developing their own reflective practice [11]. The usefulness of SPs and role-playing in SBT continues to be discussed, but there is strong argument for its application in producing well-rounded clinical practitioners, which should be the goal of every ultrasound training program [12,13,14].

### 2.1. Development of a Simulation-Based Program

The development of an SBT program may differ depending on numerous factors but should include extensive intentional planning by the education and design teams. It is often misunderstood that simply acquiring manikins (the higher the fidelity, the better) is assumed to be superior, and using them to demonstrate what was taught in class is sufficient. However, an effective SBT program is much more complex and has numerous steps, all revolving around the needs of the educational facility and the intersection points of the SBT program with the learners’ journey [15,16].

In higher education, simulation considerations often focus on the needs of a training program as interpreted by educators within that program, aiming to supplement operations within an already established educational institution [9]. However, a successful SBT program will be more easily realized if institutional realities (such as practical and organizational conditions) are considered at the earliest possible stage of planning. The scope of fiscal resources, depth of human capital expertise, and the availability of equipment and maintenance services are all issues that must be addressed early in the process.

Consideration must be given to whether the training institution has a goal of establishing a simulation center, or if the simple incorporation of simulated activities in various didactic sessions is enough. For the purposes of this paper, we focus on the latter, as the former is complex and would be best addressed when given its own platform.

**Figure 1:**
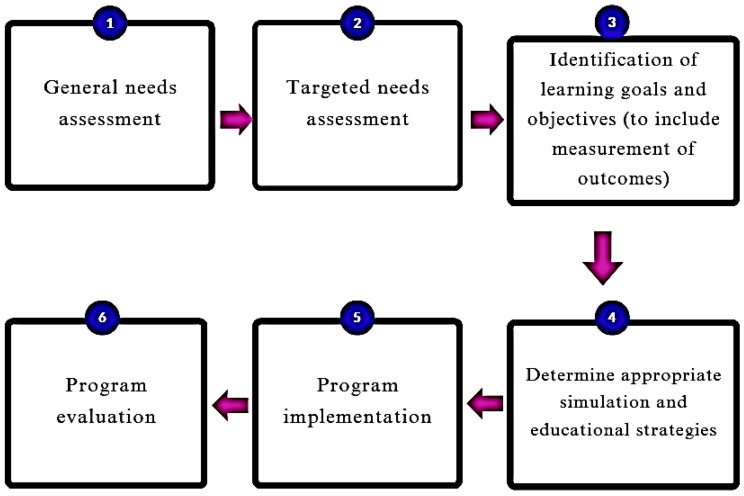
A visual representation of a six-step simulation curriculum development approach adapted from Pedersen et al. [17].

Figure 1 summarizes a six-step curriculum development approach to simulation-based programming that was presented by Pedersen et al., 2024 [17]. Though this model was presented for use in abdominal ultrasound training, its generality allows for application in the development of an Ob/Gyn curriculum as well.

### 2.2. Simulation in Obstetrics and Gynecology

Obstetrics and gynecology (OB/Gyn) is a specialty area of medical practice focusing on women’s and reproductive health. The effectiveness of OB/Gyn care regimes is greatly increased when ultrasound is used as a complement to physical exams [18].

The combination of transabdominal (TA) and endovaginal (EV) ultrasound remains a critical component of gynecologic care and has different levels of applicability in obstetric scenarios. However, the ethical boundaries of ultrasound training programs restrict the learners’ ability to practice EV and obstetric ultrasound exams outside of a diagnostic medical facility, on actual patients, and for the express purpose of providing answers to legitimate clinical questions. The challenge therefore is to design and provide opportunities for students to continuously achieve level-appropriate competence in these areas of their practice where opportunity for safe practice is severely limited.

### 2.3. Simulation Faculty Development

An effective and sustainable simulation-based training program requires the inclusion of faculty that is trained in administering an ultrasound simulation training program [19]. In technical specialties such as sonography, faculty members are often clinical experts who transition into teaching roles with limited or no formal training in education [20]. It is therefore within the institution’s best interest to have a faculty development plan that equips these instructors with the necessary skillset.

**Figure 2:**
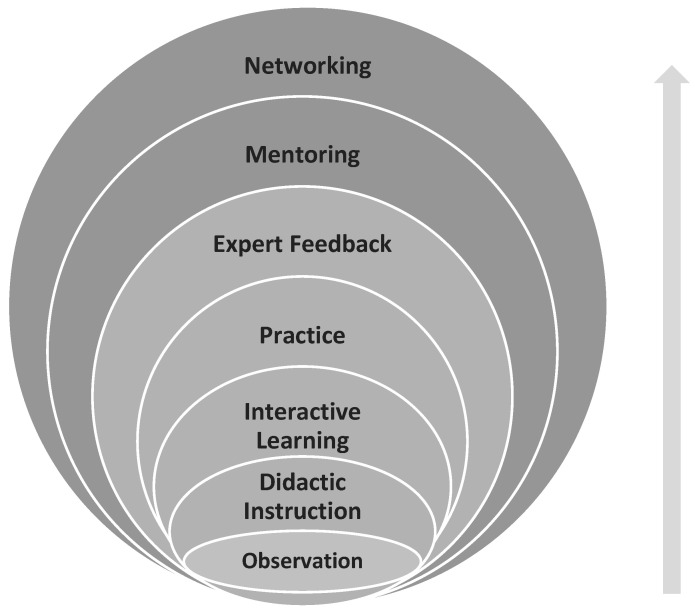
The visual representation of a seven-element tiered approach to simulation faculty development adapted from Peterson et al. [22].

There are several conventional approaches for faculty training that are widely used in higher education. Workshops, online or in-person training courses, individual assistance, and accessing communities of practice are examples of such training methods [21]. However, a tiered approach that supports progressive skill development is presented by Peterson et al. [22] and is a promising method of sustainably upskilling simulation faculty. The seven elements of this tiered approach interact in ways that provide incremental exposure to core concepts, while allowing time for these teachings to be internalized by the participating faculty. These seven elements may be divided into 2 categories, where the first five are more passive in nature and are focused inwardly on the learner. The last two elements require the active participation of the learner as they seek to learn from experts in their communities of practice. Figure 2 illustrates this relationship of these seven elements.

The process begins with the faculty member’s observation of simulation activities, leading him or her to critically analyze different ways of conducting various exercises while inspiring self-reflection. This serves as a reference point for the ensuing short didactic presentation that highlights key concepts in SBT. Through group work and collaborative exercises, faculty can then begin to work on the planning, designing, and implementing of specific simulation exercises. Ongoing practice of what is learned in the previous elements, combined with the instruction and advice from feedback received, encourages eventual mastery of techniques. The mentoring of faculty is an important step that should not be overlooked. The application of techniques that would have been learned up to this point is often learned through mentorship, as it is through this design that general skills development occurs [23].

Acknowledging the specialized skillset that is needed by simulation faculty [19] reinforces the need for planned development after these team members have been recruited. The devising of such a plan should not be seen by program leadership as optional. The appropriate training of faculty members who will be leading student instruction is as important as having proper simulation equipment in the institution’s centers of learning.

### 2.4. Equipment for Use in Gynecologic Simulation

The mastery of any sonographic technique is directly proportional to the amount of practice that is dedicated to that specific technique. A sentiment that is also frequently shared within the sonographic professional circle is that “if you don’t use it, you’ll lose it”, referring to scanning skills. With that in mind, well-designed and structured sessions provide opportunities to practice at the learner’s preferred time during their training.

To provide gynecologic scanning practice, some ultrasound programs require female students to take turns assuming the roles of patients. These students would do the necessary physical preparation of appropriately filling their urinary bladder and allow their assigned lab partner(s) to practice TA scanning on them. With biological male students being anatomically unable to act as patient models in gynecologic lab sessions, they present complexities in this lab design. This is a challenge that is commonly mitigated through proper scheduling and rotation arrangements that sees female students, or other female volunteers from outside of the program, taking the place of male students in lab sessions. Regardless of the circumstance, conducting EV scanning in such a setting is considered unethical and should be avoided. This dynamic, therefore, requires the use of simulation if students are to be appropriately trained in EV scanning technique outside of a clinical environment.

**Figure 3:**
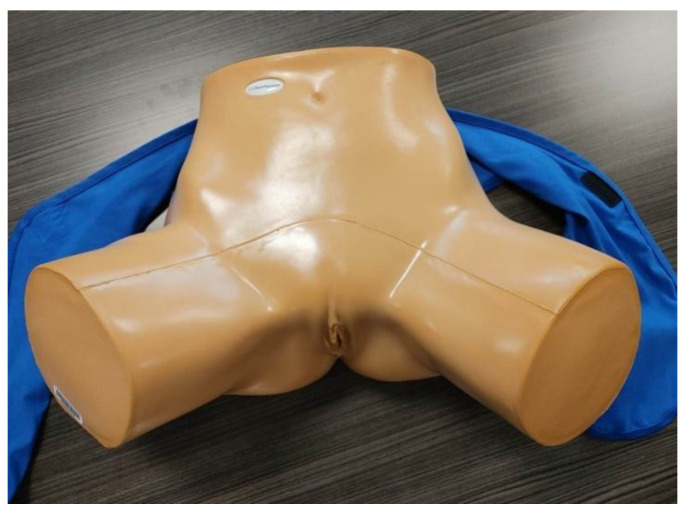
An example of a simple physical phantom (SPP) for endovaginal (EV) scanning.

As technology advances, several options for EV simulation have become available. Simple physical phantoms (SPPs) that contain replicas of bone, tissue, and organs, are available in various sizes, designs, and complexity (see Figure 3). Learners can become familiar with best practices that aid in the visualization of specific regions of the pelvis such as the adnexa and the cul de sac. The development of these skills is critical, as the importance of EV ultrasound scanning continues to be shown in clinical practice [24].

**Figure 4:**
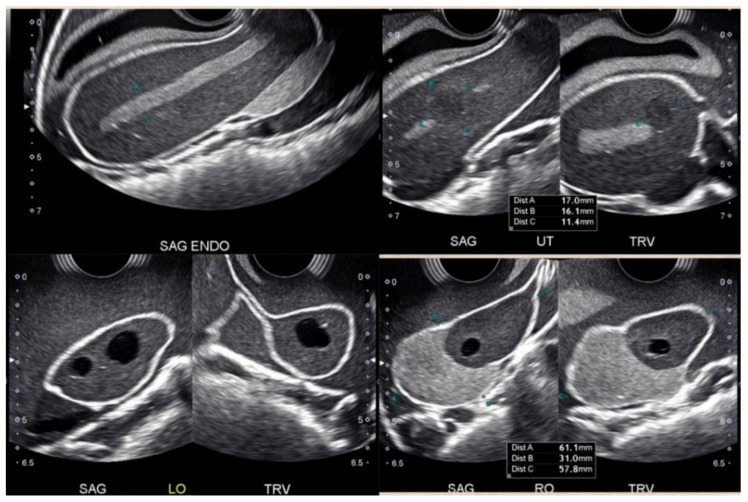
A collage showing images from a simple physical phantom (SPP) for endovaginal (EV) scanning: the endometrial stripe (**top left**), uterine fibroid (**top right**), normal ovary (**bottom left**), and ovarian pathology (**bottom right**).

Such types of phantoms introduce students to the identification, measurement, and interrogation techniques associated with anatomical structures such as the endometrium, the uterus, and the ovaries (Figure 4). Common pathologies like uterine fibroids and ovarian masses can also be visualized in some of these simulation models. Although these kinds of SPPs can be sourced with relative ease, each model will have its pros and cons and programs will need to choose based on their intended application.

Computer-based simulators (CBSs) are also an option for EV simulation. However, the range of CBS products that exist for EV scanning is less than what is available for abdominal ultrasound training. With EV scanning being intracavitary, it is difficult to create or replicate that technique in web-based applications (apps), virtual reality, tablets/phones, etc. In the realm of CBSs for EV scanning, complete systems are desirable but will often have a higher price when compared to other options. These complete systems will have a manikin that has pre-programmed software and is connected to a screen that generates images produced by a transducer that is specifically designed for use with that system. Being computer-based, these simulators are inherently able to expose students to a much wider array of pathologies and anatomical variations than the more statically designed SPPs. A summary table of pros and cons for different types of simulators is presented as Table 1.

## 3. Equipment for Use in Obstetric Simulation

Unlike with gynecologic training where students practice on each other or on volunteers during lab sessions, it is unlikely that the learners will have any hands-on experience with obstetric scanning before entering the clinic. This reality requires ultrasound programs to be even more creative in designing obstetric learning opportunities for their learners. The complexity is increased when the unique technical nuances are applied for the different stages and trimesters of pregnancy (knobology, machine program settings, application of scanning angles, etc.), and the nearly overwhelming number of pathologies that are encountered in obstetric scanning. Learning proper approach and adaptive techniques when scanning an active fetus requires interaction with real patients where students can experience the unpredictable and dynamic nature of obstetric scanning. However, there are different types of simulators that can effectively introduce learners to these concepts and provide some preparation for clinical practice.

### 3.1. First-Trimester Simulation

**Figure 5:**
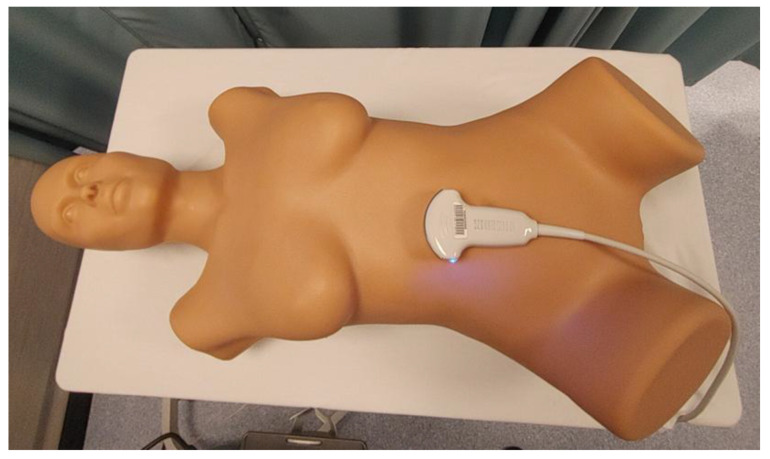
An example of a computer-based simulators (CBS) for first- and second-trimester scanning.

CBSs work well for first-trimester scanning. In these scenarios, pregnant manikins (Figure 5) will have several programs that demonstrate a pregnancy at different developmental stages in the first trimester, along with some common first-trimester pathologies.

**Figure 6:**
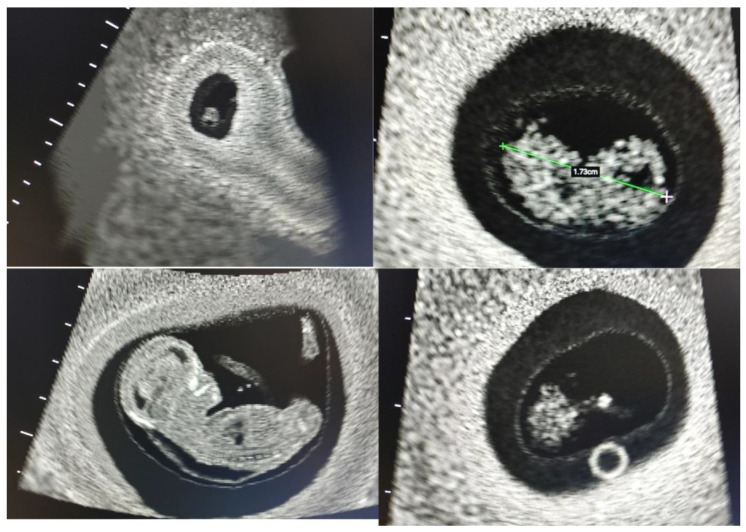
A collage showing first trimester images from a transabdominal (TA) scan on a computer-based simulators (CBS): an early gestational sac (**top left**), crown–rump length measurement of an embryo (**top right**), mid-sagittal image of a fetus (**bottom left**), yolk sac in early gestation (**bottom right**).

Learners can, at this stage, learn to locate the yolk sac, practice achieving the longest axis of an embryo or fetus, measuring the crown–rump length, assess the early placenta and the gravid uterus, identify subchorionic bleeds, locate ectopic pregnancies, and much more. This type of CBS will typically have high usability for the training program, as a single manikin can be programmed with multiple scenarios to introduce scanning considerations for multiple stages of pregnancy. Examples of images that may be acquired from a first-trimester CBS are seen in Figure 6.

First-trimester SPP options also exist and are excellent for introductory demonstrations. However, one major limitation in these situations is the limited number of images that can possibly be acquired. These types of simulators have a high chance of being quickly mastered by learners who can memorize the anatomical design of the manikin and instinctively acquire study images.

### 3.2. Second- and Third-Trimester Simulation

**Figure 7:**
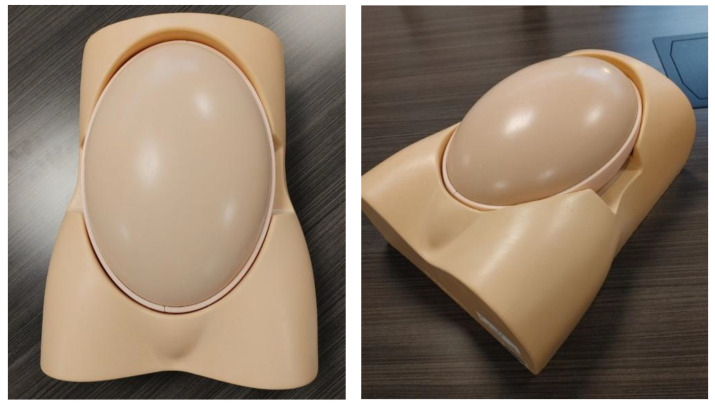
Example of second-trimester ultrasound Simple physical phantom (SPP) viewed from two perspectives.

At this stage of pregnancy, SPP and CBS options are available for training. However, SPPs can enjoy a much greater level of usability at this level with some creative planning by the lab or by the course instructor. With the focus of second-trimester simulation being mostly on determining fetal position, the techniques used in the identification of image planes, and measuring of fetal parts, SPPs offer high degree of flexibility and usability. For example, an instructor can place a manikin on the scanning bed, cover it with a towel or a sheet, and have the learner scan under the cover. The learner will, in this scenario, learn to critically analyze the images that are present on the ultrasound screen and thereby develop their spatial interpretation abilities. Some manikins have a modular design that allows the fetal position to be switched around, encouraging learners to apply even more critical thinking to their practice (Figure 7).

**Figure 8:**
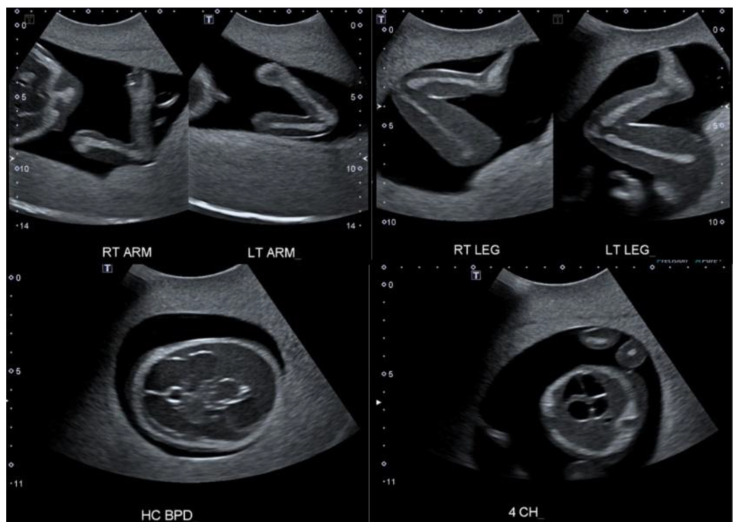
A collage showing images from a second trimester SPP for OB scanning: the upper limbs (**top left**), lower limbs (**top right**), an axial image of the brain at biparietal diameter plane (**bottom left**), and the four-chamber heart view (**bottom right**).

Along with spatial interpretation, these SPPs also allow learners to begin understanding how to manipulate the angle of insonation to achieve specific fetal images. The proper demonstration of the bones of a limb, for example, can be practiced using these simulators, and a worthwhile introduction to acquiring fetal biometric measurements can also be had. A collage of such images is shown in Figure 8.

**Figure 9:**
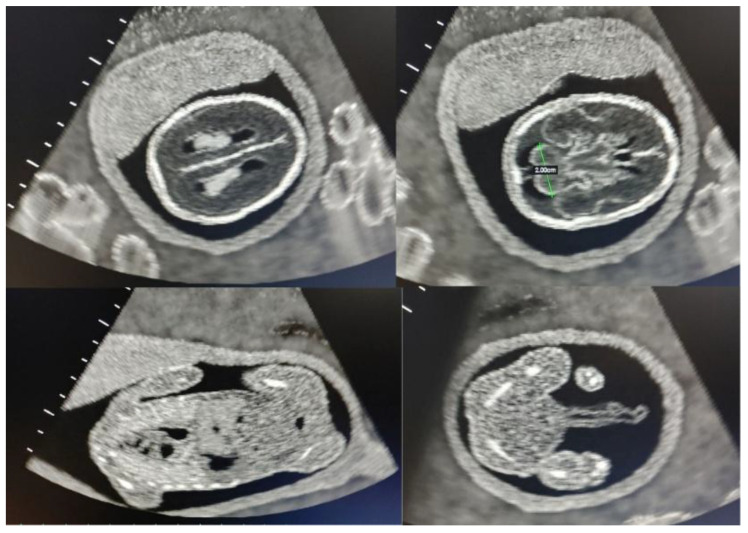
A collage showing second-trimester images from a transabdominal (TA) scan on a computer-based simulators (CBS): axial images of the choroid plexus (**top left**), an axial measurement of the cerebellum (**top right**), a coronal image of the fetal thorax and abdomen (**bottom left**), and an axial image of the abdomen at the level of the umbilical cord insertion (**bottom right**).

A CBS provides similar opportunities but with more options, given its programmability. The fetal position can be changed, challenging the learner to apply spatial reasoning and adjust scanning techniques. One advantage of CBS is its ability to replicate several pathologies through its programming. By leveraging this capability, instructors can help students learn how to interact with common pathologies before they enter clinical practice, thereby increasing their ability to detect and effectively image these pathologies in real patients [25]. CBSs are also well-suited to instruction relating to the detailed second-trimester ultrasound exam, as they create opportunities to introduce a plethora of pathologies for fetal anomaly screening that are not typically available in SPPs. Facial clefts, spinal pathology, and limb deformities are often available for demonstration in these simulators and provide students an invaluable introduction to the component of practice where scan routines need to be adapted to navigate these encounters. Examples of images acquired from a second-trimester CBS are presented in Figure 9.

## 4. Simulation for Non-Technical Skill Development

The term ‘non-technical skills’ as applied in healthcare is

“*the ‘cognitive (situation awareness and decision making), social (teamworking, leadership, and communication) and personal resource (managing stress and fatigue) skills that complement technical skills and contribute to safe and efficient task performance by individuals…*”.

[5]

Healthcare professionals must demonstrate competence extended beyond the confines of technical skills. To support the development of safe and inclusive communities, training programs have become more intentional about developing the non-technical skills of their learners [26,27]. Sonographers, as clinicians, are also called upon to improve their person-centered practice, and this training needs to begin as early as possible.

Obstetric and gynecologic studies are among the more delicate clinical scenarios to maneuver in ultrasound practice. Not only must patients accommodate and endure examinations of a sensitive nature (as in the case of gynecology), but there is an exponential increase in emotional investments when dealing with obstetric studies. The practicing professional must be ready to handle these scenarios which may include correct and responsive communication (both verbally and non-verbally), the accurate interpretation of the patients’ body language, or the effective handling of undesirable examination outcomes. These are all essential skills that can often mean the difference between a satisfied patient, or complaints and legal claims. Creating scenarios for learners to develop interpersonal and non-technical skills should not be an after-thought or treated as a nice-to-do. Instead, training programs should be prepared to invest heavily in ensuring the availability of a robust learning opportunity to that end.

Perhaps the simplest way to implement this training is to employ SPs. Not only is the validity of their inclusion established, but their incorporation in training regimes and other teaching and evaluating exercises are acceptably reliable [28]. In these situations, learners can interact with designed, scripted, and controlled interactions that mimic real-world situations. Being in a safe and controlled environment that allows for guidance, debriefing, and repeated practice encourages deep and lasting learning, which will give the learners confidence to bring their skills into clinical practice.

Recently, virtual reality and gamified training options have also been introduced [29]. In the case of virtual reality, learners wear headsets and use peripheral connections to access pre-programmed training modules that are meant to replicate real-life interpersonal interactions. Gamification provides similar opportunities, but it may be argued the applicability of both options is reduced as learners advance through their studies, making them ideal introductory tools but potentially less effective for senior learners. Though these options are well-aligned with intentions to afford controlled training opportunities, they can be cost-prohibitive, and their overall effectiveness is still being studied [29].

## 5. Discussion and Future Aspects

Healthcare training programs remain in delicate circumstances. It is well accepted that clinical placements enhance critical learning [17,30], but the availability of clinical sites continues to be a challenge. As the state of healthcare continues to be discussed globally, one of the deficits that is frequently mentioned is the unavailability of sufficient practitioners in the various fields to adequately provide needed services [19,31,32,33]. The mass retirement of healthcare professionals, high staff turnover, and widespread burnout all contribute to this situation. One response to that challenge is to increase the number of training opportunities, with the intention of producing more graduates to fill the gap. Unfortunately, there is likely to be a related increase in the competition for the limited number of existing clinical sites. We have discussed the role of simulation in OB/Gyn, but there is room for further application of this importantly versatile tool. There is both a need and an opportunity for exploration of the use of simulation in the assessment of learner competency. With proper design, implementation, and control, some aspects of simulation (including those discussed in this paper) could be used for more than just formative training and evaluation. Although simulation is inferior to training in a clinical setting [34], it may be able to provide summative information on student readiness for sonographic practice. There is a need for more studies in this area.

Ultrasound simulators represent a significant financial investment for institutions, and the value and benefit of this investment must be justified. When considering the long-term benefits of educational efficacy and cost-effectiveness, however, these expenses may prove to be advantageous for the learners, the institution, and ultimately for the patients. Toolsgaard et al. [35] have demonstrated that applying a cost-effectiveness model can link the quality of clinical care to training-related costs. Further investigation into the extension of this premise to sonographic training may be needed.

Lastly, research in previous years has confirmed the prevalence of repetitive strain injuries (RSIs) in sonographers [17,26]. Though RSIs are typically attributed to prolonged scanning in improper or awkward positions [17], less is known about whether learners are being prematurely exposed to such risks through the use of SPP and CBS units. Although these simulators and phantoms are constructed with tissue-like polymers, they are incapable of completely mimicking human anatomy and are usually a lot more rigid. Therefore, there is a requirement for increased scanning pressure when using these units, which could be seen as tributary to RSIs [36]. An exploration into the issue of safety in relation to design and usage of simulators and phantoms is indeed indicated.

## 6. Conclusions

Simulation has become an essential element in OB/Gyn ultrasound education, becoming an expectation for ultrasound training programs. By investigating current best practice, we have shown educators the wide variety of options to support and design an effective simulation program for OB/gyn sonography. Today, institutes are encouraged to explore the various options and approaches that can be applied to sonographic training in a simulated environment, recognizing the potential for students to develop technical and non-technical skills in these scenarios.

## Figures and Tables

**Table 1:** General pros and cons for different types of simulators.

Type of Simulator	Pros	Cons
Computer-Based Simulator	Capable of demonstrating many different pathologies	Expensive to purchase and maintain
	Software versions or updates allow for programing to match student’s progress	Requires dedicated space in classroom or lab
	Can provide exposure to different types of anatomical variations	Cumbersome and difficult to transport
		Delicate/Fragile
Simple Physical Phantoms	Relatively inexpensive	Instructional effectiveness is reduced as students progress in their training
	Easily transported and stores	Limited number of pathologies or anatomical variation possible
	Ideal introductory tools for new learners	

## Data Availability

Data are contained within the article.
